# The Emergence of Tool Use in Preterm Infants

**DOI:** 10.3389/fpsyg.2016.01104

**Published:** 2016-07-19

**Authors:** Maja Petkovic, Lauriane Rat-Fischer, Jacqueline Fagard

**Affiliations:** Laboratoire Psychologie de la Perception, Centre Biomédical des Saints-Pères, CNRS UMR 8158, Université Paris DescartesParis, France

**Keywords:** preterm infants, visual-manual coordination, tool-use

## Abstract

Preterm born children without neurological impairments have been shown to present some visual-manual coordination deficits, more or less depending on their tonicity and the degree of prematurity. In this paper, we compare the development of tool use in 15–23-month-old preterm infants born after 33–36 weeks of gestation without neurological complications with that of full-term infants according to corrected age. Understanding the affordance of a tool is an important cognitive milestone in early sensorimotor period. Using a tool to bring within reach an out-of-reach object, for instance, has been shown to develop during the 2nd year in full-term infants. Here we presented preterm infants with an attractive toy out of reach and with a rake-like tool within reach in five conditions of spatial relationships between the toy and the tool. Like full-terms, preterm infants used the tool with success in conditions of spatial contiguity around 15–17 months. In conditions of a spatial gap between tool and toy, i.e., the only conditions which shows without ambiguity that the infant understands the affordance of the tool, preterm infants as a group showed no delay for tool use: the frequency of spontaneous successes started to increase after 18 months, and demonstration became effective after that age. However, further analyses showed that only the preterm infants without hypotonia and born after 36 weeks of pregnancy developed tool use without delay. Hypotonic preterm infants were still largely unsuccessful in the conditions of spatial gap, even at the end of the study. The degree of prematurity also influenced the performance at tool use. These results, following the observation of a delay in the development of bimanual coordination and of handedness in the same infants at 10–12 months in a previous study, show that low risk preterm infants can still be impaired for the development of new manual skills beyond the 1st year. Thus, hypotonic preterm infants and infants born before 36 weeks of pregnancy should be followed and might benefit from early intervention programs.

## Introduction

The World Health Organization (WHO) defines preterm infants as those born before 37 weeks of completed pregnancy. Unfortunately, the prevalence of developmental disabilities in preterm children is still high and many children born preterm present mild dysfunctions such as learning disabilities, lower IQ scores and specific cognitive deficits affecting attention, such as ADHD (attention-deficit/hyperactivity disorder), as well as visual function, visual-motor integration, and executive functions (Van Braeckel et al., 2010; Baron et al., 2011; Bos and Roze, 2011). The causes of these disabilities are still unclear, although they are sometimes connected with neurological impairments such as intraventricular hemorrhage (IVH) and are often related to decreasing gestational age at birth (Moster et al., 2008; Volpe, 2009). However, there is a growing body of cross-sectional and longitudinal research showing that even preterm infants without major neurological complications (e.g., such as IVH grades III and IV, congenital abnormalities, etc.), considered “healthy” or “low risk”, have poorer cognitive and neuropsychological performances than full-term infants in preschool and school age period (Vicari et al., 2004; Linnet et al., 2006; Sansavini et al., 2011; Caravale et al., 2012; Nepomnyaschy et al., 2012). It is yet unknown to what extent later cognitive deficits could be predicted earlier in infancy. We thus decided to test low risk preterm infants for the acquisition of tool use, which is an important cognitive milestone in infants’ early sensorimotor period. Before describing what is known about the emergence of tool use in full-term infants, we will briefly summarize the main disabilities reported for these low risk preterm children.

**Cognitive performances** have been well studied in low risk preterm children, in preschool and school-age period (Vicari et al., 2004; Linnet et al., 2006; Sansavini et al., 2010, 2011, 2014; Caravale et al., 2012; Nepomnyaschy et al., 2012). For instance, Caravale et al. (2005, 2012) observed lower neuropsychological performances in low risk preterm infants at 3–4 years, some of them still present at 5 years. They found that 3-year-old and 4-year-old preterm children, when compared to full-terms, achieved lower mean scores on the *Stanford-Binet intelligence scale, visual perception test* (observation of geometric shape), *visual-motor integration test* (paper-pencil components of the Visual-Motor Integration Test), *memory for location test* and *sustained attention.* When tested at 5 years, preterm children still obtained significantly lower mean scores than full-terms on *visual-motor integration test* and *visual perception.*

Much less studies on sensorimotor development have focussed on low risk preterm infants within the first 2 years of life. Regarding **motor development**, results from kinematics studies showed some motor impairment during reaching in 6- to 7-month-old preterm infants, with lower mean and final velocities when compared to full-term infants (Rönnqvist and Domellöf, 2006; Toledo and Tudella, 2008). However, some studies found that the reaching development of 8-, 10-, and 12-month-old preterm infants was similar to that of their full-term peers when corrected age was used (see for review O’Sullivan et al., 2012).

Whether or not preterm infants have normal **visual function** is an important question when considering visual-manual development. Visual attention as assessed through fixation shifts is less mature in preterm than in full-term infants (Ricci et al., 2010). Similarly, preterm infants have a lower proportion of smooth pursuit eye movements (Grönqvist et al., 2011), lower visual recognition memory (Rose et al., 2001), and narrower visual field (Petkovic et al., 2016). Some studies aimed at disentangling the influence of visual versus motor deficits resulting in the visual-motor impairment in preterm children. And according to Teplin et al. (1991) and Goyen et al. (2008), preterm infants visual-manual deficits are more due to a fine motor deficit than to low visual perception.

Tool use is one of the **visual-manual skills** which develop during the 2nd year of life. It is one of the hallmarks of the sensorimotor period (Rat-Fischer et al., 2012). Tool use belongs to the category of means-end behaviors, for which reaching a goal requires an intermediate action. For instance, when an interesting object is placed at the end of an uninteresting one, such as an out-of-reach toy at the end of a string or placed on a cloth within reach, infants must discover that pulling the string (or the cloth) will allow them to retrieve the toy. Such means-end behaviors develop between 7 and 12 months (Piaget, 1936; Fagard, 1998; Willatts, 1999). When the uninteresting object within reach is not connected to the interesting object, the problem is much harder for the infant. For instance, Bates et al. (1980) compared 9-10-month-old infants retrieving an out-of-reach toy placed either on a cloth, at the end of a string, or at different positions near three kinds of utensils likely to help the children to retrieve it (hoop, crook, or stick). The children succeeded in the conditions where toy and means to retrieve it were physically linked (‘unbreakable contact”, cf. means-end situations just mentioned) but less often when the contact was breakable, and not at all in the condition without any contact.

When the object within reach is not physically attached to the toy, a rigid object is required to bring the toy closer. The rigid object must be used as a tool. One widely accepted definition of a tool is an object independent from the object on which it is supposed to act. Infants’ first tool is generally the spoon. Infants often start using a spoon or trying to use a spoon around the end of the 1st year. Note that the spoon is a particular tool, in that prior to using the spoon themselves, infants have many opportunities to see their family and other people using a spoon to eat.

Using unfamiliar tools to bring an out-of-reach object within reach is succeeded later. A few studies have focussed on how infants learn to use such a new tool (see Keen, 2011 and Greif and Needham, 2011, for reviews). Many of them have focussed on perceptual factors, all stressing that difficulty increases with increasing spatial gap between the tool and the object to be acted upon (Bates et al., 1980; van Leeuwen et al., 1994), and more generally with an increasing number of steps needed to achieve the required result (Smitsman and Cox, 2008). Other studies aimed at finding the mechanisms leading to learning the affordance of a tool and its skillful use, namely trial and error, observational learning, and practice (Rat-Fischer et al., 2012; Esseily et al., 2013, 2016; Fagard et al., 2014, 2016; Somogyi et al., 2015, for a review). These studies showed that it is not before the end of the 2nd year that infants become successful in using tools when there is a spatial gap between them and the target, implying a true understanding of the tool’s function. Indeed, discovering how to use an unfamiliar tool represents a very complex affordance learning: the infant must combine several affordances such as grasping the tool, placing it behind the toy, pulling it toward himself, but he must also anticipate the state of the rake after being moved. This makes tool use an important hallmark of cognitive development, from an evolutionary as well as a developmental point of view. Since, as we saw previously, some preterm children exhibit cognitive deficits in school years, it would be interesting to know whether they show an earlier typical development of tool use or not.

So far, there have been no studies carried out on the development of tool use in preterm infants. Investigating the development of this cognitively demanding visual-manual skill could bring us closer to evaluating the relationship between visual-manual early development and possible later cognitive deficits seen in preterm children. Since such relationships between early visual-motor development and later cognitive abilities have been observed in full-terms (see for instance, Piek et al., 2008), we might hypothesize that tool-use could be a sensitive tool for detecting future cognitive deficits.

## Materials and Methods

### Participants

Twelve preterm infants (eight girls and four boys) participated in this study. All infants included in the study were considered healthy, with no IVH above grade I, no evidence of visual or auditory impairment, and no major neurological complications (see **Table 1**). They were part of a longitudinal study which started at 5 months with a visual evaluation and an evaluation every month for a visual-manual follow-up (see Petkovic et al., 2016). The visual evaluation showed no visual impairment in any infant, except a tendency toward narrower visual field in preterm infants compared to full-terms. Preterm infants were recruited from a database of the Zagreb Special Hospital for Children with Neurodevelopmental and Motor Disorders. After approval of the Hospital Ethical Committee, the families were sent an information leaflet about the study. The families who expressed interest in taking part in the study signed a parental consent form.

**Table 1:** Medical information about preterm infants.

Type of pregnancy:	Single birth: 4	Twin birth: 8 (4 × 2)
Category of gestational age at birth:	Moderate preterm birth: 5 33 weeks: 2 (1 hypotonic) 34 weeks: 3 (1 hypotonic)	Late preterm birth: 7 36 weeks: 7 (1 hypotonic)
Category of birth weight:	Normal birth weight: 6	Low birth weight: 6
Incubator:	Yes: 7	No: 5
Muscle tone:	Hypertonic: 10 (including 3 moderate)	Hypotonic: 3 (including 2 moderate)
IVH:	IVH, grade I: 6	No: 6
Type of birth:	Vaginal: 5	Cesarean: 7
Follow-up by physician:	Yes: 12	No: 0
VAT (Visual evoked potentials):	No visual disabilities: 12	Visual disabilities: 0
BERA	No auditory disabilities: 12	Auditory disabilities: 0

The preterm infants were compared to 60 full-term infants (20 girls and 40 boys). These full-term infants were part of a previous cross-sectional study (Rat-Fischer et al., 2012), and constituted the control group of the present study in which the authors of the first study actively participated, thus guarantying a common methodology. Five age groups of 12 participants were part of the control group: 14-month-olds (13 months 28 days to 14 months 13 days), 16-month-olds (15 months 28 days to 16 months 9 days), 18-month-olds (17 months 26 days to 18 months 4 days), 20-month-olds (19 months 27 days to 20 months 10 days), and 22-month-olds (21 months 25 days to 22 months 5 days). Infants were recruited from a list of local families who expressed interest in taking part in studies of infant development. Prior parental consent was granted before observing the infants.

For tool use the preterm infants were tested every 2 months from 15 to 23 months, thus at 15, 17, 19, 21, and 23 months. We chose to test them at these ages so that we could compare them to the full-terms tested at 14, 16, 18, 20, and 22 months. Thus, to correct for prematurity, the preterm infants were compared with full-terms a month younger.

### Procedure

**FIGURE 1:**
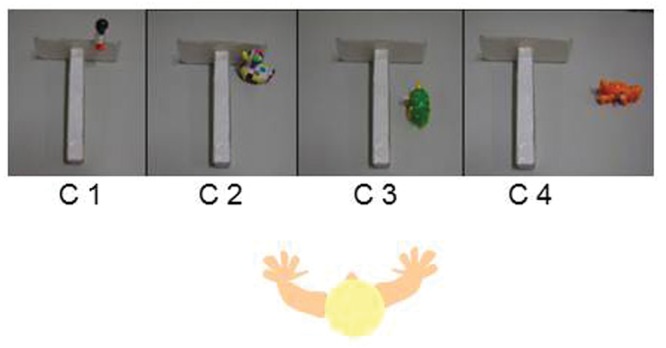
**Tool use conditions (C5, where the tool is handled to the child) is not shown**.

The experimental apparatus was designed to assess at what age and in which conditions infants were capable of using a tool to retrieve an out-of-reach toy. The desired toy was placed out of reach at different positions near a white cardboard rake-like tool designed to be visually plain (see **Figure 1**). During the whole experiment, infants sat on the lap of one of their parents in front of a table. The experimenter sat facing the infants across the table. A digital video camera recorded the whole session.

After the infants were familiarized with the surroundings and with the material, an attractive toy was placed in front of them successively in five conditions: toy attached to the rake part of the tool (C1: no spatial gap, attached), toy inside and against the rake part of the tool (C2: no spatial gap, unattached), toy inside the tool but not against it (C3: small spatial gap), toy to the side of the tool (C4: large spatial gap), and toy in the middle of the table with the tool directly held out to the infant by the experimenter (C5: effectively a very large spatial gap). The conditions were presented in order of increasing spatial gap from C1 to C5. All infants received one trial at C1, where they all immediately succeeded. They were then directly presented with two trials at C2. If both trials were successful, they received two trials at C3 (and so on until C5). If infants failed to one or both trials of a condition, they were given one or two additional trials of that condition. If infants failed to retrieve the toy in two of three trials, the parents or the experimenter gave two consecutive demonstrations of the failed condition. If infants failed in a condition after a demonstration, they were directly presented with the C5 condition. Thus, the C3 and C4 conditions were presented only if infants succeeded in the previous condition either spontaneously or after a demonstration; only the C1, C2, and C5 conditions were presented to all infants.

### Data Analysis

A trial was coded both for global success/failure to retrieve the toy in a given condition, and on the basis of a behavioral category evaluated for each trial. When an infant succeeded at least at two trials in a given condition, he was coded as successful for this condition. When an infant succeeded at one trial but failed at the other two trials, he was coded as failure for this condition but he was granted 33.3% successful trials. For the behavioral category a score of 0 was attributed when infants expressed no interest in the toy, the tool, or (more generally) the task; a score of 1 was attributed when infants were mostly interested in the out-of-reach toy, pointing toward it and possibly trying to retrieve it without using the tool; a score of 2 was attributed when infants were mainly interested in manipulating the tool itself, possibly alternating their attention between the toy and the tool but not in connecting them; a score of 3 was attributed when infants systematically and repetitively brought the tool to bear on the toy but seemingly not with the purpose of retrieving the toy; a score of 4 was attributed when infants brought the tool to bear on the toy obviously with the purpose of retrieving the toy but failed. A score of 5 was attributed when infants succeeded in retrieving the toy with the tool.

The percentage of success was calculated in two ways: 1% of infants with success for the condition (success if the infant had been successful at both consecutive trials or at 2/3 of the trials of the condition); 2/ mean percentage of successful trials for the condition. When infants were not tested for the following condition after repeated failure at the easier preceding condition, they were given a score of 0.

A Friedman ANOVA was calculated to test the effect of age and condition on the percentage of successful trials, separately for preterm children and full-terms. We did not include C1 in these Friedman ANOVAs since there was not enough variance for this condition. A Mann–Whitney test was used to compare the preterm children with the full-terms, and within the preterm children, to compare the children as a function of their tonicity, degree of prematurity, and birth weight. To evaluate the impact of observation of a demonstration, we calculated an ANOVA for Group (x 2; Preterm infants, Full-terms), for Condition (x 2, Before Demo, After Demo, repeated measures) for each age separately.

## Results

### Spontaneous Success

**FIGURE 2:**
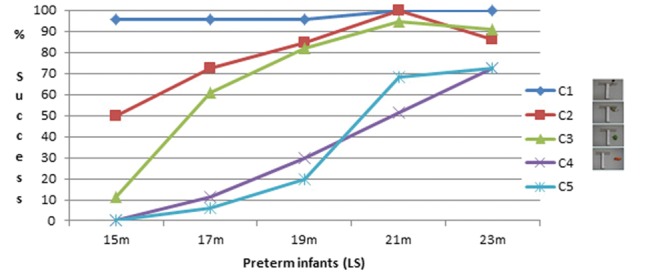
**Percentage of successful infants (Preterm infants)**.

**FIGURE 3:**
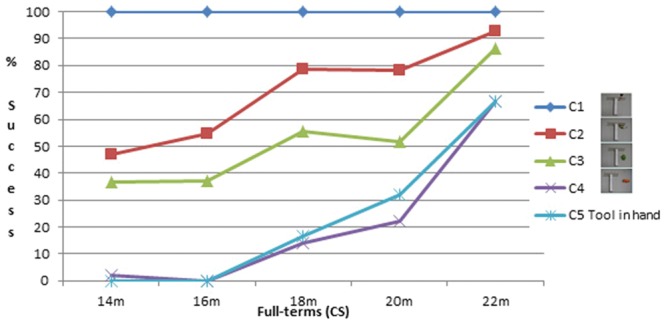
**Percentage of successful infants (Full-terms)**.

Percentage of successful infants is shown on **Figures 2** and **3**. C1 was almost always successfully performed from the first session in both groups. In both groups there was a decrease in the percentage of successful infants from C1 to C2 and to C3. There was a clear decrease in the percentage of successful infants for the two most difficult conditions, C4 and C5, which did not differ much from each other.

For the preterm infants, a Friedman ANOVA was calculated on the percentage of successful trials for each condition as a function of Age. It shows a near-significant age effect for C2, *F*(10,4) = 8.5; *p* = 0.075; a significant age effect for C3, *F*(10,4) = 27.3; *p* = 0.00002; for C4, *F*(10,4) = 15.8; *p* = 0.003, and for C5, *F*(10,4) = 23.9; *p* = 0.00008. We also checked whether the conditions significantly differed for each age. A Friedman ANOVA indicates a significant difference for Condition at 15 months, *F*(12,3) = 17.7; *p* = 0.0005; at 17 months, *F*(11,3) = 20.9; *p* = 0.0001; at 19 months, *F*(10,3) = 19.2; *p* = 0.0002, and at 21 months, *F*(11,3) = 12.6; *p* = 0.006, but not at 23 months (*p* = 0.17).

Similarly, for full-terms, a Friedman ANOVA was calculated for each condition as a function of Age on the percentage of successful trials. It shows a significant age effect for C2, *F*(12,4) = 16.2; *p* = 0.003; for C3, *F*(11,4) = 12.4; *p* = 0.015, and for C5, *F*(8,4) = 15.8; *p* = 0.003. The effect does not reach significance for C4, *p* = 0.10. We also checked whether the conditions significantly differed for each age. A Friedman ANOVA indicates a significant difference for Condition at 14 months, *F*(11,3) = 17.9; *p* = 0.0005; at 16 months, *F*(11,3) = 18; *p* = 0.0004; at 18 months, *F*(10,3) = 18.5; *p* = 0.0003, and at 22 months, *F*(12,3) = 12.8; *p* = 0.005, but not at 20 months, *p* = 0.22.

A Mann–Whitney was calculated to check whether the group of preterm infants differed from the full-terms. At 15–14 months, 17–16 months, 19–18 months, and 23–22 months, there was no significant difference between the two groups for any of the conditions; at 21 months the preterm infants were significantly more performant than the 20-month-old full-terms at C2, *z* = 2.6, *p* = 0.008, and C3, *z* = 0.9, *p* = 0.049. There was no significant difference at C4 and C5 between both groups at any age.

### The Influence of Demonstration at C4 and C5

**FIGURE 4:**
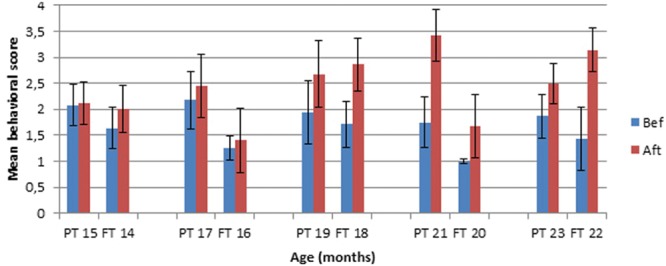
**Mean behavioral score at C4–C5 Before versus After demonstration in Preterm (PT) and Full-term (FT) infants**.

Infants who did not spontaneously succeed were given a demonstration of how to use the tool. We analyzed the effect of the demonstration for C4 and C5 only, which are the main conditions of interest, the only ones which cannot be succeeded by chance thanks to the spatial contingency between the tool and the toy. We compared the mean behavioral score before and after the demonstration at C4 and C5 combined. Before 19–18 months, there was no effect of the demonstration (see **Figure 4**).

We calculated an ANOVA for Group (x 2; Preterm infants, Full-terms) and for Condition (x 2, Before Demo, After Demo, repeated measures) for each age separately. At 15–14 months, there is no effect of Group, no effect of Condition, and no Group × Condition interaction. At 17–16 months, there is no effect of Condition but there is a significant effect of Group, *F*(1,17) = 5.07, *p* = 0.04. The group of preterm infants had a higher mean score than the group of full-terms both before and after demonstration. There is no Group × Condition interaction. At 19–18 months, there is no effect of Group but there is an effect of Condition, *F*(1,13) = 8.98, *p* = 0.01. Infants had a higher behavioral score after than before demonstration. There is no Group × Condition interaction. At 21–20 months, there is an effect of Condition, *F*(1,12) = 12.8, *p* = 0.004. In addition, there is an effect of Group, *F*(1,12) = 10.8, *p* = 0.01. The group of preterm infants had a higher mean behavioral score than the group of full-terms, and infants from both groups had a higher score after than before demonstration. There is no Group × Condition interaction. At 23–22 months, there is an effect of Condition, *F*(1,9) = 8.6, *p* = 0.017, but no effect of Group and no Group × Condition interaction. In both groups infants had a higher behavioral score after than before demonstration. Thus, starting at 19–18 months, the effect of Condition is significant. Even though there is a Group effect at some ages, the complete absence of significant Group × Condition interaction indicates a comparable effect of demonstration for both groups.

### Preterm Infants’ Performance as a Function of their Characteristics (Muscle Tone, Prematurity, Birthweight)

We checked whether tool-use performance varied with muscle tone (hypertonia vs. hypotonia), number of weeks of pregnancy (33, 34, or 36 weeks), and birth weight (normal vs. low) within the group of preterm infants. Globally, the three hypotonic infants were less successful at tool use than the nine hypertonic infants (see **Table 2**). A Mann–Whitney test calculated on the percentage of success at all conditions with muscle tone as independant variable shows no significant differences between both groups at 15 and 17 months. At 19 months, the hypertonic infants were significantly more successfull than the hypotonic ones for C2 only, *z* = -3.3, *p* = 0.001. At 21 months, the hypertonic infants were significantly more successful than the hypotonic infants for C5, *z* = -2.2, *p* = 0.029, and almost significantly so for C3, *z* = -1.8, *p* = 0.06. At 23 months, the hypertonic infants performed significantly better than the hypotonic ones for all conditions; C2, *z* = -1.9, *p* = 0.05; C3, *z* = -2.6, *p* = 0.01; C4, *z* = -2.5, *p* = 0.01; C5, *z* = -3, *p* = 0.002.

**Table 2:** Mean percentage of success among premature children as a function of muscle tone and age.

	C2	C3	C4	C5
**15 m**				
hypo	33,33	11,11	0,00	0,00
hyper	55,56	11,11	0,00	2,22
**17 m**				
hypo	66,67	44,45	0,00	2.23
hyper	74,07	66,67	14,81	7,41
**19 m**				
hypo	44,45	66,67	10	6.7
hyper	100,00	87,50	38.9	28.7
**21 m**				
hypo	100,00	62.5	17	22.7
hyper	100,00	96,30	62,96	83,33
**23 m**				
hypo	55,56	63,89	24.2	0,00
hyper	96,30	100,00	88,89	96,30

The five infants born after 33 or 34 weeks (moderate preterm) were less successful than the seven infants born after 36 weeks (late preterms) but only at the two most difficult conditions (C4 and C5) of tool use (see **Table 3**). A Mann–Whitney test calculated on the percentage of success at all conditions as a function of Prematurity shows that the two groups displayed no significant difference at 15 and 17 months. At 19 months, the late perterm infants performed significantly better than the moderate preterms for C4, *z* = -2.28, *p* = 0.022, and C5, *z* = -1.94, *p* = 0.05. At 21 months, the late preterm infants were almost significantly more successful than the moderate preterms for C4, *z* = -1.83, *p* = 0.06, but significance is not reached for C5, *p* = 0.12. At 23 months, the late perterm infants performed almost significantly better than the moderate preterms for C4, *z* = -1.85, *p* = 0.06, but non-significantly better for C5, *p* = 0.14. There was no difference in performance according to birthweight.

**Table 3:** Mean percentage of success among premature children as a function of prematurity and age.

	C2	C3	C4	C5
**15 m**				
moderate	73,30	19,90	0,00	0,00
late	33,30	4,80	0,00	2,90
**17 m**				
moderate	86,70	66,70	20,00	13,30
late	61,90	57,10	4,8	0,90
**19 m**				
moderate	86,70	80,00	0,00	0,00
late	85,70	85,70	54,30	38,60
**21 m**				
moderate	100,00	95,00	20,00	40,00
late	100,00	82,70	73,90	88,30
**23 m**				
moderate	80,00	93,30	40,00	53,30
late	90,50	89,30	96,10	85,70

## Discussion

The preterm infants were first successful at the C2 and C3 conditions (both conditions with no spatial gap or with a small spatial gap), with an increase in the frequency of success from 15 to 21 months when almost all infants were successful. However, in the C2 and C3 conditions, the toy was positioned so that it laid in the trajectory between the tool head and the infants. Thus, simply pulling the tool through a small distance would inevitably bring the toy into reach. Thus, in these conditions, successes could have been achieved by chance, at least at the early sessions, because infants could pull the tool and obtain the toy by pure spatial contingency. High percentages of success in the C2 and C3 conditions with little or no spatial gap, therefore, should not be considered as true indicators of infants’ comprehension of the tool. In contrast C4 and C5 (both conditions with a large spatial gap) could barely be succeeded by chance. Because of the spatial gap between the tool and the toy in these conditions, infants must understand the usefulness of the tool to succeed. Success at C4 and C5 occurred much later during development than success at C2 and C3. First spontaneous successes arose toward the end of the 2nd year.

These results are close to the results of the full-terms, obtained in a previous study and used here as controls for the premature children (Rat-Fischer et al., 2012). Preterm infants were compared with full-terms 1 month younger for correction of prematurity. The percentage of successful infants tends to be slightly higher in the preterm infants than in the full-terms. However, this difference between the two groups is significant only for C2 and C3 and only at 21–20 months. There is no significant difference in the percentage of success at C4 and C5 between the two groups. This result indicates that, as a group, the preterm infants are not delayed in their acquisition of tool use. The tendency for them to perform even better at the easiest conditions can be explained by the fact that there was a correction for prematurity: it could be that at that age, the time spent after birth is more important than the total number of months since conception. Another reason for the better performance of the preterm infants, not exclusive of the first one, is that the preterm infants were tested longitudinally and therefore had the opportunity to practice, whereas the full-terms were seen only once. This is in line with the difference that we observed between a cross-sectional and a longitudinal study, the infants from the latter being slightly more advanced than the infants from the former (Rat-Fischer et al., 2012; Fagard et al., 2014). To control for this possibility, we also compared the preterm infants with the full-terms of our longitudinal study carried out on five children from 12 to 18 months (Fagard et al., 2014). At the ages when such comparison was possible (15, 17, and 19 months for the preterm children, compared with the full-terms at 14, 16, and 18 months, respectively), we observed no significant group differences. This confirms that, as a group, the preterm children of our study are not delayed in their acquisition of tool use. We did not report a qualitative analysis of the strategies used by the preterm children because we observed the same fluctuations and the same lack of tendencies in the strategies used before being successful as reported in the previous studies with full-terms (Rat-Fischer et al., 2012; Fagard et al., 2014): infants sometimes beg for the toy, sometimes play with the tool, sharing their attention between both, until they become able to spread their attention simultaneously on the toy and the tool and to make the link between them.

When the infants failed, we gave them two demonstrations. The effect of the demonstration on the performance was analyzed for C4 and C5, by comparing the mean behavioral level of performance on trials before demonstration and after demonstration. In preterm infants, there was no effect of the demonstration before 19 months: starting at that age infants tended to have a higher mean behavioral score after demonstration. These results are close to those found with full-terms who start to score better after demonstration than before at 18 months. The lack of interaction between Group and Condition reveals that preterm infants are able to learn from observation of a model to the same extent and at the same age (corrected for the preterm infants) as the full-terms.

Finally we checked whether, beyond the absence of group differences between the preterm infants and the full-terms, we would find differences within the group of preterm infants depending on muscle tone, degree of prematurity and birth weight. Muscle tone was clearly associated with a lower performance within preterm infants. Whenever there was a difference between hypotonic and hypertonic preterm infants, the latter were always more successful than the former. And at 23 months hypotonic preterm infants were significantly less successful than hypertonic infants in all conditions. It is worth noting that at 23 months of age, the hypotonic children still showed an important difference in percentage of success between the two easiest conditions (C2 and C3) and the two most difficult ones (C4 and C5) whereas hypertonic children were mostly successful at all conditions, including the two conditions with a large gap between the tool and the toy. These results are in continuity with our observation of the same infants at 6 to 12 months: hypotonic infants showed significantly less visual-manual coordination on the Peabody Developmental Motor Scale than preterm infants with high muscle tone (hypertonia) at all ages. In addition, hypotonia was also significantly associated with lower bimanual coordination at 11- and 12-months postnatal age. We suggested that these results could be interpreted with reference to the development of the cerebellum, which is involved in dynamic feedforward motor control, and has been found to be impaired in preterm infants (Allin et al., 2004; Limperopoulos et al., 2005). The cerebellum is important in muscle tone regulation (Shah et al., 2006), which could explain why muscle tone modulated performance in our first study (Petkovic et al., 2016). But the cerebellum is also involved in cognitive skills, as shown by child (Berger et al., 2005) and adult studies (Timmann and Daum, 2007). This could also explain the persistent effect of muscle tone in the two most cognitive conditions of tool use at the end of the 2nd year.

The degree of prematurity also influenced tool use performance at C4 and C5, but not in the easiest conditions. The percentage of successful trials at C4 and C5 was significantly lower in moderate compared to late preterm infants at 19 months, when the latter started to succeed in these conditions. The tendency of the late preterm infants to show more success than the moderate preterm infants in these conditions could still be observed at up to 23 months, the oldest age in our study. This indicates that late preterm infants start earlier to show some successful trials than moderate preterm infants in the two most cognitively demanding conditions. In our first longitudinal study, gestational age at birth similarly influenced the age of emergence of bimanual coordination. In addition, the two 33-week preterm infants had a very low laterality index compared to the 34- and 36-week preterm infants up to 12 months. Thus, the degree of prematurity partly accounted for the preterm group’s results on the emergence of a new skill (bimanual coordination) or of new movement organization (having a preferred hand). Tool use is also a new skill for infants in their 2nd year of life: it is worth noting that we also observe a delay in the emergence of this skill in moderate preterm infants. Similar findings on the effect of the degree of prematurity on movement organization have been reported in other studies (Domellöf et al., 2011). Finally, birth weight did not significantly influence the tool use results, in the same way it did not influence any of the results of the first longitudinal study.

## Conclusion

Our results indicate that, as a group, the preterm infants seem to have a normal development of tool use. However, not all preterm infants developed tool use without delay. Hypotonic and moderate preterm children seemed delayed in their acquisition of tool use, in the same vein as they showed a delay in the acquisition of bimanual coordination and of handedness during their 1st year. By following these hypotonic low risk preterm children, it should be possible to evaluate which of the observed delays in these two studies are the best markers of later cognitive deficits in childhood. It could then lead us to evaluate when and how to help hypotonic and moderate preterm children in order to prevent later cognitive deficits.

However, these results must be taken with care because there are several limitations in our study. First the results bear on a small number of children. In particular, even though all hypotonic infants were clearly delayed in their acquisition of tool use, they were only three in this study. Similarly, the moderate preterm children are only five, and two of them are also hypotonic. A replication of the study with a larger sample of children is clearly needed to draw firm conclusions. In addition, the corrected age might have over-compensated the prematurity and a comparison for chronological age would be welcome. It might reveal a delay in the preterm children considered as a group that was not revealed by a corrected age comparison; however, the delay observed for hypotonic children would be even more striking in a chronological comparison. Finally, it would be important to test the visual field of the premature children when they start being tested for tool use. In our first study, the hypotonic preterm children showed a narrower visual field than the hypertonic children at the age of 5 months (Petkovic et al., 2016). Preterm children were not tested again for vision in this study: it is not excluded that a narrower visual field partly explains the hypotonic preterm children’s delay in the emergence of tool use.

## Author Contributions

MP and JF contributed to this work by planning, organizing, and caring the follow-up study. Analyzing the data and interpreting the results in terms of current theories of action-perception approach and development of preterm infants. LR-F. contributed to this work by taking part in preparation of the methodology referring to her own research on tool us carried out in full-term infants. Also by reflecting on the results found in this study to her own findings on full-term infants.

## Conflict of Interest Statement

The authors declare that the research was conducted in the absence of any commercial or financial relationships that could be construed as a potential conflict of interest.

The reviewer CR and handling Editor declared their shared affiliation, and the handling Editor states that the process nevertheless met the standards of a fair and objective review.
